# Effects of ruminal lipopolysaccharides on growth and fermentation end products of pure cultured bacteria

**DOI:** 10.1038/s41598-022-20073-2

**Published:** 2022-09-23

**Authors:** Efstathios Sarmikasoglou, Jessica Ferrell, James R. Vinyard, Michael D. Flythe, Apichai Tuanyok, Antonio P. Faciola

**Affiliations:** 1 Department of Animal Sciences, University of Florida, Gainesville, FL 32611 USA; 2 United States Department of Agriculture, Agricultural Research Service, Lexington, KY 40546 USA; 3 Department of Infectious Diseases and Immunology, College of Veterinary Medicine, University of Florida, Gainesville, FL 32611 USA

**Keywords:** Applied microbiology, Bacteria, Microbial communities, Industrial microbiology

## Abstract

Elevated levels of ruminal lipopolysaccharides (LPS) have been linked to ruminal acidosis; however, they result in reduced endotoxicity compared to LPS derived from species like *Escherichia coli*. Additionally, there is a knowledge gap on the potential effect of LPS derived from ruminal microbiome on ruminal bacteria species whose abundance is associated with ruminal acidosis. The objective of this study was to evaluate the effects of LPS-free anaerobic water (CTRL), *E. coli*-LPS (E. COLI), ruminal-LPS (RUM), and a 1:1 mixture of *E. coli* and ruminal-LPS (MIX) on the growth characteristics and fermentation end products of lactate-producing bacteria (*Streptococcus bovis* JB1, *Selenomonas ruminantium* HD4) and lactate-utilizing bacterium (*Megasphaera elsdenii* T81). The growth characteristics were predicted based on the logistic growth model, the ammonia concentration was determined by the phenolic acid/hypochlorite method and organic acids were analyzed with high performance liquid chromatography. Results indicate that, compared to the CTRL, the maximum specific growth rate of *S. bovis* JB1 decreased by approximately 19% and 23% when RUM and MIX were dosed, respectively. In addition, acetate and lactate concentrations in *Se. ruminantium* HD4 were reduced by approximately 30% and 18%; respectively, in response to MIX dosing. Compared to CTRL, lactate concentration from *S. bovis* JB1 was reduced approximately by 31% and 22% in response to RUM and MIX dosing; respectively. In summary, RUM decreased the growth and lactate production of some lactate-producing bacteria, potentially mitigating the development of subacute ruminal acidosis by restricting lactate availability to some lactate-utilizing bacteria that metabolize lactate into VFAs thus further contributing to the development of acidosis. Also, RUM did not affect *Megasphaera elsdenii* T81 growth.

## Introduction

Gram-negative bacterial cell membranes contain lipopolysaccharide (LPS), a tripartite molecule composed of: a lipid A moiety, a core oligosaccharide region, and repeating O-antigen units^1^. Lipid A moiety consists of acyl chains, whose length is associated with the ability of the molecule to trigger the host immune response^2,3^. In general, lipid A moieties with six acyl chains (e.g. *Escherichia coli* lipid A) induce strong host immunological response, whereas, the under-acylated forms exhibit weak host immunological response^4^. Several diseases are associated with increased levels of LPS in blood plasma, including ruminal acidosis in cattle^5^. Ruminal acidosis is a metabolic disorder that occurs when the consumption of rapidly fermentable carbohydrates replace effective fiber, causing excessive organic acid (volatile fatty acids and lactate) accumulation in the rumen, which has been associated with reduced feed intake, reduced milk production, and milk fat depression^6,7^. In general, ruminal pH of 5.6 or below is considered a threshold for ruminal acidosis, where subacute acidosis is characterized with a pH between 5 and 5.6 and acute acidosis is characterized when ruminal pH is below 5. In subacute acidosis, the driving factor that decreases ruminal pH is the accumulation of volatile fatty acids and their decreased absorption from the ruminal epithelium. Lactic acid is produced by lactate-producing bacteria and is normally rapidly metabolized into volatile fatty acids, as long as the pH remains above 5 (subacute acidosis)^5,8^. Thus, SARA may be a consequence of VFA accumulation, and not necessarily lactate accumulation; however, lactate can be converted to VFA and indirectly contribute to SARA by contributing to VFA accumulation. When pH drops below 5 for a sustained period, the growth of lactate-utilizing bacteria is inhibited and lactic acid accumulates, characterizing acute acidosis^5^.

Ruminal bacteria composition is determined by several factors, including the diet^9^. In general, cows fed high forage diets contain more Gram-negative bacteria, whereas cows fed high grain diets contain more Gram-positive bacteria^9^. In the rumen Gram-negative bacteria are the major source of LPS^10,11^. The presence of LPS in the ruminal fluid is normal since bacterial death and lysis are normal processes that take place during ruminal fermentation; however, under SARA conditions, ruminal LPS concentration is much greater compared to healthy cattle^12,13^. Additionally, LPS seems to affect ruminal fermentation and bacterial diversity by stimulating the growth of Gram-negative bacteria associated with starch digestion^11^. Furthermore, from previous reports, LPS from *Escherichia coli* (*E. coli*) has been suggested to be utilized as a substrate for acidosis related bacteria including *Streptococcus bovis* and *Selenomonas ruminantium*^14^ therefore, LPS seems to have a potential effect on the growth of bacteria related to acidosis.

Previously, two studies have shown that LPS affect the growth of *Anaeroplasma abactoclasticum* (*An. abactoclasticum*) strain 6-1^15^ and that seems to work as a factor to stimulate the growth of *S. bovis* JB1^14^. However, the underlying mechanism of that response is still unknown. In previous studies, *E. coli* O25:B6 LPS was extracted with Boivin^16^ and Westphal^17^ methods and included into *An. abactoclasticum* strain 6-1 media. The LPS extracted by the Boivin method stimulated the growth of strain 6-1, whereas the LPS extracted by the Westphal method inhibited the growth of strain 6-1. The Boivin method, which uses trichloroacetic acid extraction, yielded LPS contaminated with residual peptides that are removed in the Westphal method, which uses phenol extraction. Peptides from the Boivin method could have acted as growth promoters and consequently stimulated the growth of strain 6-1, while the residual phenol from the Westphal preparation could have inhibited the growth of strain 6-1^15^. In a more recent study, it was hypothesized that the polysaccharidic part of LPS would be the stimulatory factor for *S. bovis* JB1^14^. For this reason, delipidated LPS, which contains mostly the polysaccharidic part, was dosed into *S. bovis* JB1 culture; however, the stimulatory effect was reduced compared to the regular LPS (polysaccharidic and lipid A part). Thus, it was inferred that the stimulatory effect of LPS could be associated with the lipid A region.

Although the mechanism of action of LPS on the growth of ruminal bacteria is not fully understood, LPS effect is evident. Previous reports have shown that *E. coli* O25:B6 LPS stimulated and inhibited the growth of *An. abactoclasticum* strain 6-1, when extracted with Boivin and Westphal methods, respectively^15^. In contrast, *E. coli* O111:B4 LPS stimulated the growth of some lactate producing bacteria and did not affect lactate utilizing bacteria^14^. In addition, similarly to Robinson’s findings^15^, a previous case report indicated that ruminal-LPS is not structurally equivalent to *E. coli*-LPS, primarily, because the former exhibits under-acylated (low endotoxic) and the latter hexa-acylated (high endotoxic) lipid A structures^18^, which could potentially be linked with their ability to stimulate the growth of ruminal bacteria^14^.

We hypothesized that ruminal-LPS would stimulate the growth of pure ruminal bacteria cultures that utilize lactate (*Megasphaera elsdenii* T81) and slow the growth of pure ruminal bacteria cultures (*Selenomonas ruminantium* HD4, *Streptococcus bovis* JB1) contributing to the development of ruminal acidosis. Therefore, we aimed to evaluate the effect of ruminal-and *E. coli*-LPS on the growth of lactate-producing bacteria (*Selenomonas ruminantium* HD4, *Streptococcus bovis* JB1) and lactate-utilizing bacteria (*Megasphaera elsdenii* T81), as well as assess any potential effect of their combination.

## Results

### Effect of LPS on bacterial growth

**Figure 1:**
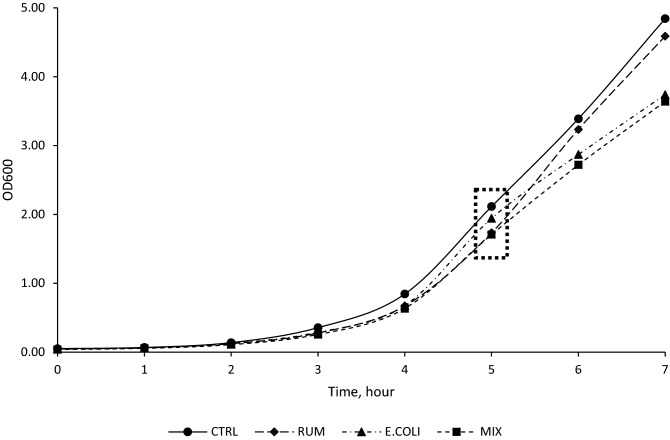
Effects of ruminal, *E. coli*, and Mix (1:1) LPS on lag time and maximum specific growth rate of *Selenomonas ruminantium* HD4. Dotted box = Samples collected at mid-exponential phase for NH_3_-N, and organic acids concentration determination. *CTRL* control group (LPS-free anaerobic water), *RUM* ruminal LPS (200,000 EU), *E. COLI E. coli* LPS (200,000 EU), *MIX* 1:1 E. coli: Ruminal LPS (200,000 EU).

**Figure 2:**
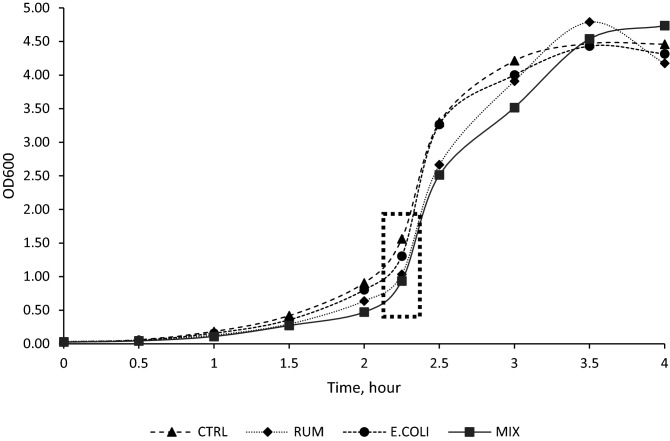
Effects of ruminal, *E. coli*, and Mix (1:1) LPS on lag time and maximum specific growth rate of *Streptococcus bovis* JB1. Dotted box = Samples collected at mid-exponential phase for NH_3_-N, and organic acids concentration determination. *CTRL* control group (LPS-free anaerobic water), *RUM* ruminal LPS (200,000 EU), *E. COLI E. coli* LPS (200,000 EU), *MIX* 1:1 *E. coli*: Ruminal LPS (200,000 EU).

**Figure 3:**
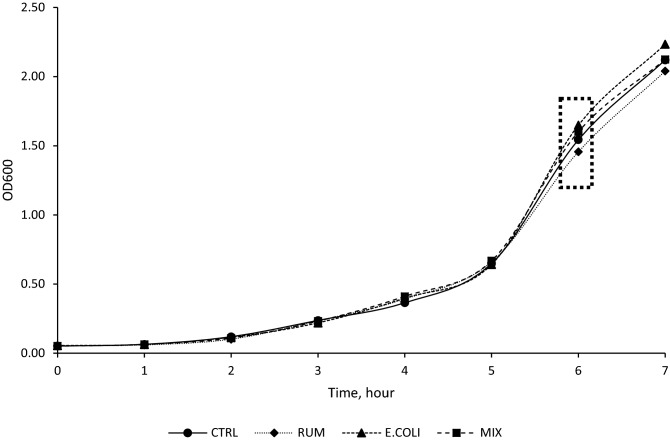
Effects of ruminal, *E. coli*, and Mix (1:1) LPS on lag time and maximum specific growth rate of *Megasphaera elsdenii* T81. Dotted box = Samples collected at mid-exponential phase for NH_3_-N, and organic acids concentration determination. *CTRL* control group (LPS-free anaerobic water), *RUM* ruminal LPS (200,000 EU), *E. COLI E. coli* LPS (200,000 EU), *MIX* 1:1 E. coli: Ruminal LPS (200,000 EU).

We dosed 200,000 EU of *E. coli*-LPS, ruminal-LPS and MIX (1:1)-LPS to three different ruminal bacterial species in pure culture. Regarding *Se. ruminantium* HD4 growth characteristics, there were no differences observed for initial OD (Y_0_), real OD (Yt), the change of OD from OD_0_ to OD_t_ (C), the lag, and the maximum specific growth rate (Table 1; Fig. 1). Regarding the growth characteristics of *S. bovis* JB1, no effect was observed for Y_0_; however, Yt was reduced by 48.4% (*P* < 0.01) and 29.7% (*P* < 0.01) in response to RUM and MIX LPS, respectively, when compared to CTRL. In addition, Yt was reduced by 41.3% (*P* < 0.01) and 20.0% (*P* < 0.01) in response to RUM and MIX LPS, respectively, when compared to E. COLI. Also, Yt was reduced by 36.2% (*P* < 0.01) in response to RUM when compared to MIX treatment. The C was reduced by 19.6% (*P* = 0.03) and 23.9% (*P* = 0.03) in response to RUM and MIX LPS, respectively, when compared to CTRL. In addition, compared to the E.COLI, the C was reduced by 18.8% (*P* = 0.03) and 23.1% (*P* = 0.03) in response to RUM and MIX LPS, respectively. The lag time was increased by 16.4% (*P* = 0.02) in response to MIX LPS when compared to the CTRL. Also, the lag time was reduced by 6.7% (*P* = 0.02) and 3.5% (*P* = 0.02) in response to E. COLI and RUM dosing respectively, when compared to the MIX LPS treatment (Table 2; Fig. 2). Compared to CTRL, the maximum specific growth rate of *S. bovis* JB1 was reduced by 19.1% (*P* = 0.03) and 23.5% (*P* = 0.03) in response to RUM- and MIX-LPS dosing, respectively (Table 2). Also, compared to E. COLI, the maximum specific growth rate was reduced by 18.5% (*P* = 0.03) and 22.9% (*P* = 0.03) in response to RUM and MIX dosing, respectively. No effects were observed for Y_0_, Yt, C, lag and maximum specific growth phase of *M. elsdenii* T81 in response to any of the treatments (Table 3; Fig. 3).

**Table 1:** Effects of ruminal, *E. coli*, and Mix (1:1) LPS on lag time, maximum specific growth rate, ammonia nitrogen, and OAs concentration of *Selenomonas ruminantium* HD4.

Item^2^	Treatment^1^				SEM	*P*-values
	CTRL	RUM	E. COLI	MIX		
Y0	0.06	0.06	0.05	0.06	0.01	0.21
Y	0.81	0.73	0.64	0.61	0.05	0.06
C	2.05	1.68	1.88	1.66	0.32	0.52
Lag, min	194.72	166.52	208.80	200.61	24.57	0.39
μmax, h^−^1	0.42	0.35	0.39	0.34	0.06	0.51
Total OAs, mM	11.03^a^	10.33^ab^	9.80^bc^	9.12^c^	0.45	0.02
**Individual OA, mM**						
Formate	–	–	–	–		
Acetate	0.87^a^	0.76^ab^	0.73^bc^	0.61^b^	0.05	0.01
Propionate	1.52	1.47	1.39	1.37	0.08	0.23
Butyrate	–	–	–	–		
Lactate	8.49^a^	7.94^ab^	7.53^bc^	6.98^b^	0.36	0.03
Succinate	0.15	0.16	0.15	0.16	0.36	0.99
Valerate	–	–	–	–		
N-NH_3_, mM	7.77	8.18	8.01	7.91	0.76	0.29

The lag time and the maximum specific growth rate were calculated based on the prediction from logistic function.

^a-c^Means within the same row with different superscripts differ (*P* ≤ 0.05).

^1^Experimental treatments: *CTRL* control group (LPS-free anaerobic water), *RUM* ruminal LPS (200,000 EU), *E. COLI* E. coli LPS (200,000 EU), *MIX* 1:1 E. coli: Ruminal LPS (200,000 EU).

^2^Items: Y0 is initial OD0; Y is real ODt; C is change of OD from OD0 to ODt, lag = lag time; μmax = maximum specific growth rate; Total OAs = total organic acids; Individual OA = individual OAs detected; N-NH_3_ = ammonia nitrogen.

**Table 2:** Effects of ruminal, *E. coli*, and Mix (1:1) LPS on lag time, maximum specific growth rate, ammonia nitrogen, and OAs concentration of *Streptococcus bovis* JB1.

Item^2^	Treatment^1^				SEM	*P*-values
	CTRL	RUM	E. COLI	Mix		
Y0	0.03	0.03	0.03	0.03	0.01	0.22
Y	0.91^a^	0.64^b^	0.80^a^	0.47^c^	0.05	< 0.01
C	3.27^a^	2.63^b^	3.24^a^	2.49^b^	0.38	0.03
Lag, min	112.72^a^	120.24^a^	116.7^a^	131.22^b^	7.84	0.02
μmax, h^−1^	1.36^a^	1.10^b^	1.35^ac^	1.04^b^	0.07	0.03
Total OAs, mM	18.58^a^	12.34^b^	16.70^ab^	14.02^bc^	0.83	0.01
**Individual OA, mM**						
Formate	0.59	–	0.48	–	0.05	0.24
Acetate	0.29	0.23	0.26	0.22	0.02	0.18
Propionate	–	–	–	–		
Butyrate	–	–	–	–		
Lactate	17.70^a^	12.10^b^	15.96^ab^	13.80^bc^	0.78	0.01
Succinate	–	–	–	–		
Valerate	–	–	–	–		
Ethanol	1.02	0.56	0.66	0.50	0.30	0.63
N-NH_3_, mM	8.42	8.77	8.65	8.61	0.23	0.37

The lag time and the maximum specific growth rate were calculated based on the prediction from logistic function.

^a–c^Means within the same row with different superscripts differ (*P* ≤ 0.05).

^1^Experimental treatments: *CTRL* control group (LPS-free anaerobic water), *RUM* ruminal LPS (200,000 EU), *E. COLI* E. coli LPS (200,000 EU), *MIX* 1:1 *E. coli*: Ruminal LPS (200,000 EU).

^2^Items: Y0 is initial OD0; Y is real ODt; C is change of OD from OD0 to ODt; *Lag* lag time, *μmax* maximum specific growth rate, *Total OAs* total organic acids; *Individual OA* individual OAs detected, *N-NH*_*3*_ ammonia nitrogen.

**Table 3:** Effects of ruminal, *E. coli*, and Mix (1:1) LPS on lag time, maximum specific growth rate, ammonia nitrogen, and OAs concentration of *Megasphaera elsdenii* T81.

Item^2^	Treatment^1^				SEM	*P*-values
	CTRL	RUM	E. COLI	MIX		
Y0	0.05	0.06	0.06	0.05	0.01	0.78
Y	0.65	0.66	0.64	0.67	0.04	0.87
C	1.49	1.40	1.59	1.55	0.35	0.62
Lag, min	203.84	201.12	222.24	206.37	38.00	0.63
μmax, h^−1^	0.26	0.24	0.28	0.27	0.02	0.63
Total OAs, mM	17.32	16.67	17.69	18.48	1.04	0.24
**Individual OA, mM**						
Formate	–	–	–	–		
Acetate	7.10	6.64	7.25	7.31	0.20	0.28
Propionate	8.61	8.26	8.65	9.49	0.35	0.28
Butyrate	1.05	1.20	1.15	1.11	0.05	0.77
Lactate^3^	–	–	–	–		
Succinate	–	–	–	–		
Valerate	0.56	0.56	0.63	0.56	0.01	0.12
N-NH_3_, mM	9.44	8.88	9.21	9.01	0.62	0.49

The lag time and the maximum specific growth rate were calculated based on the prediction from logistic function.

^1^Experimental treatments: *CTRL* control group (LPS-free anaerobic water), *RUM* ruminal LPS (200,000 EU), *E. COLI* *E. coli* LPS (200,000 EU), *MIX* 1:1 E. coli: Ruminal LPS (200,000 EU).

^2^Items: Y0 is initial OD0; Y is real ODt; C is change of OD from OD0 to ODt; *Lag* lag time, *μmax* maximum specific growth rate, *Total OAs* total organic acids, *Individual OA* individual OAs detected, *N-NH*_*3*_ ammonia nitrogen.

^3^Lactate 50 mM was utilized as a growth substrate and no additional lactate was produced by T81 strain and considered as non-detected.

### Effect of LPS on fermentation end products

Total OAs concentration from *Se. ruminantium* HD4 was decreased by 11.2% (*P* = 0.02) and 17.3% (*P* = 0.02) in response to E. COLI and MIX treatment, respectively, compared to the CTRL (Table 1). Compared to RUM, the MIX exhibited reduced total OAs concentration by 11.7% (*P* = 0.02, Table 1). The total OAs from RUM treatment was not different compared to CTRL or E. COLI (Table 1). Regarding individual OAs, acetate concentration from *Se. ruminantium* HD4 was decreased by 16.1% (*P* = 0.01) and 29.9% (*P* = 0.01) in response to E. COLI and MIX LPS, respectively, compared to the CTRL (Table 1). Acetate concentration from *Se. ruminantium* HD4 was reduced by 19.6% (*P* = 0.01) in response to MIX compared to RUM (Table 1). The lactate concentration from *Se. ruminantium* HD4 was decreased by 11.3% (*P* = 0.03) and 17.8% (*P* = 0.03) in response to E. COLI and MIX LPS dosing, respectively, compared to the CTRL (Table 1). Also for lactate concentration in *Se. ruminantium* HD4*,* there were no differences in terms of RUM treatment compared to CTRL, E.COLI or MIX and also E.COLI treatment was not different to MIX (Table 1). Propionate, succinate, and ammonia concentrations from *Se. ruminantium* HD4 were not different among treatments (Table 1). Lastly, formate, butyrate, and valerate were not detected (Table 1).

Total OAs concentration from *S. bovis* JB1 was decreased by 33.6% (*P* < 0.01), 24.5% (*P* < 0.01) in response to RUM and MIX LPS respectively, compared to CTRL. In addition, the total OAs concentration from *S. bovis* JB1 was greater (35.3%, *P* < 0.01) when E. COLI was dosed compared to RUM. Lastly, total OAs concentration from *S. bovis* JB1 tended to be reduced by 16.1% (*P* = 0.06) in response to MIX compared to E. COLI (Table 2). Lactate concentration from *S. bovis* JB1 was decreased by 31.6% (*P* = 0.01) and 22.1% (*P* = 0.01) in response to RUM and MIX LPS dosing, respectively, compared to the CTRL (Table 2). We also found that lactate production from *S. bovis* JB1 was reduced by 31.9% (*P* = 0.01) in response to RUM compared to the E. COLI dosing and tended to be reduced by 13.5% (*P* = 0.09) in response to MIX compared to E. COLI dosing (Table 2). Acetate, ethanol, and ammonia concentrations from *S. bovis* JB1 were not different among treatments (Table 2). Formate concentration from *S. bovis* JB1 was not detected (~ 0 mM) in RUM and MIX treatments, while it was detected but did not differ between CTRL and E. COLI treatments (Table 2). Lastly, propionate, butyrate, succinate and valerate were not detected (Table 2).

No effects on any of the OAs concentration of *M. elsdenii* T81 were observed in response to any of the treatments (Table 3).

## Discussion

In our study we observed that *Se. ruminantium* HD4, compared to the CTRL, had a reduction in total OAs, acetate and lactate concentration in response to E. COLI and MIX treatments. Regarding *S. bovis* JB1, we found that, compared to the CTRL, the increase of OD, the maximum specific growth rate, total OAs, and lactate concentration decreased in response to RUM and MIX LPS, respectively. Contrary to our hypothesis, not only RUM LPS but interestingly all LPS treatments resulted in reductions in maximum specific growth and increased lag time in lactate producing bacteria and/or reduced production of their respective fermentation end products.

Previously, it has been suggested that LPS from *E. coli* O25:B6 was essential for the growth of several ruminal strains of *Anaeroplasma abactoclasticum* in pure culture^15^. Although we could not find information about *E. coli* O25:B6 LPS structure, there are studies with the *E. coli* O26:B6 LPS which is similar to O25:B6. The LPS from serotype O26:B6 exhibits a short chain-length structure which is closer to the mutant rough strain LPS (only lipid A). However, Robinson et al., (1975) were not able to elucidate the underlying mechanisms on this response. In a more recent study, it was hypothesized that the polysaccharidic part of LPS would be the stimulatory factor of lactate-producing bacteria^14^. For this reason, *S. bovis* JB1 was treated with delipidated LPS, which contains mostly the polysaccharidic part; however, the stimulatory effect was reduced compared to the regular LPS (polysaccharidic and lipid A part). Thus, it was inferred that the stimulatory effect of LPS would be associated with the lipid A part.

One possible explanation could be associated with the fatty acid composition of the respective lipid A moieties^18^. Previous findings have shown that in the absence of lipid A, the growth of lactate producing and lactate utilizing bacteria was not affected, thus indicating that the lipid A was the contributing factor associated with their growth kinetics^14^. Structurally, the lipid A is comprised of a glucosamine disaccharide backbone, which is usually phosphorylated at 1΄ and 4΄ positions of the saccharides and acylated at positions 2΄ and 3΄ of each monosaccharide portion^19,20^. Acyl chains of variable length, are directly esterified with the sugar moiety (primary acyl chains) while the secondary acyl chains form ester bonds with hydroxyl groups of primary acyl chains^21^. All primary acyl chains of *E. coli* lipid A are hydroxymyristates (saturated fatty acids with 14 carbons atoms) and one of the two secondary acyl chains is myristate (C14:0) while the other one being laurate (C12:0)^4^. However, the number, position, and length of the esterified acyl chains vary among the different bacterial species and there are no data about the fatty acid composition of pure or mixed ruminal lipid As.

Studies that have been conducted to the genus *Bacteroides* in humans, have shown that the human *Bacteroides* strains usually produce saturated or monohydroxylated heptadecanoic acid (C17:0), hexadecanoic acid (C16:0) and pentadecanoic acid (C15:0) on their respective lipid As^22,23^. Contrary to humans, ruminal *Bacteroides* have been reported to be abundant in un-weaned calves, but decreased in abundances when calves were exposed to higher starch diets post-weaning^24^. Typically, in the adult bovine rumen, the genus *Prevotella*, that belongs to the same phylum of *Bacteroides*, becomes abundant^25^. *Prevotella* in humans, has been reported to express mostly C16 and C14^26^, or C15, C16 and C17^27^; therefore, it would be possible that the mixed ruminal LPS would express mostly C15–17 fatty acids on its lipid A. Increased concentrations of the aforementioned fatty acids would have a direct antimicrobial implication to the ruminal microbiome.

In general, free fatty acids are able to disrupt the electron transport chain and oxidative phosphorylation of bacterial cell membranes^28,29^. Their mechanism of action is not specific and is attributed to interferences with the cellular energy production^30^, inhibition of enzymatic activity^31^, impaired of nutrient uptake^32^, generation of peroxidation and auto-oxidation degradation products^33^, or direct lysis of bacterial cells^34^.

In addition, all of the aforementioned fatty acids exhibit antimicrobial properties either against fungi^35^ or bacteria^36^. More specifically, myristate and laurate expressed from *E. coli* lipid A have been reported to inhibit the growth of *Listeria monocytogenes* in milk samples^37^, as well as *Clostridioides difficile *in vitro^38^, respectively. Concerning the fatty acids found in *Bacteroides*, pentadecanoic acid exhibit antifungal properties^35^ while hexadecanoic acid exhibit antibacterial functions^39^. Therefore, the inhibition of growth and decrease in production of fermentation end products could be a result of the antibacterial properties from the fatty acids in our LPS treatments. Lastly, differences were observed between different LPS treatments in which *S. bovis* JB1 reduced its total OA production on MIX treatment in greater rate than E. COLI treatment, compared to CTRL. This outcome probably indicates a potential biological interaction of MIX treatment between ruminal and *E. coli* LPS that may be associated with the combination of their fatty acids from their respective lipid A moieties. The MIX treatment was made up with a combination of ruminal and *E. coli* LPS (1:1), thus its fatty acid composition was a combination of these sources. More specifically, the E. COLI and RUM treatments were composed mostly by C12, C14, and C15–17, respectively, thus MIX treatment would be expected to have a combination of both. By accounting the fact that fatty acids have a quite broad mechanism of action against bacteria and also that the MIX treatment had a highly variable fatty acid composition, the MIX treatment could potentially disrupt the cell membrane of *S. bovis* JB1 by several different mechanisms.

Currently the underlying mechanism of how fatty acids permeate the outer cell membrane is not yet elucidated.

An interesting aspect of our results is the fact that it contradicts previous findings from our lab where *E. coli* LPS stimulated the growth of lactate producers^14^; however, it is important to note here that in this study the organisms were acquired from a different library and also the previous study was the only one investigating the effects of LPS sources in ruminal bacteria. Therefore, the novelty of this research topic and the limited number of studies (two) would not allow us to consider *E. coli* LPS as a positive control considering potential variations from different microbial collections.

On this study we evaluated some of the important species of ruminal lactate producing and utilizing bacteria; however, even at the strain level within the same species distinct growth patterns and susceptibility to LPS treatments could be observed. Therefore, in order to assess more broadly, the effects of ruminal LPS on the ruminal microbiome, future studies should be done in other ruminal lactate producers such as *Succinivibrio dextrinosolvens*, *Lactobacillus ruminis*, and ruminal lactate utilizers such as *Se. ruminantium* subs. lactilytica in pure- and co-culture level. More specifically, *Se. ruminantium* subsp. lactilytica has its own LPS gene clusters^40^ and if LPS negatively affects its growth, then it could negatively affect lactate fermentation, thus exacerbating the development of lactic acidosis. Furthermore, more research should focus on elucidating the structure and immunopotential of lipid A expressed from *Se. ruminantium* subsp. lactilytica LPS, as well as, to investigate any potential interactions between that lipid A and lipid As from sources, such as *E. coli* and *Prevotella spp.* In addition, since these bacteria have been associated with ruminal acidosis, caution should be made with extrapolations to other lactate producers and utilizers that were not tested herein.

Lastly, our results set the basis for the existence of a potential association between ruminal LPS and ruminal bacteria growth; however, some limitations that should be investigated in the future include: (1) potential adaptation of ruminal bacteria under repeated exposure to ruminal LPS in order to investigate any tolerance that these bacteria would exhibit on repeated LPS exposures, (2) potential dose effect of ruminal LPS on ruminal bacteria growth in order to establish any potential association between the development of ruminal acidosis and the ruminal LPS concentration, (3) potential LPS effects under different ruminal Ph, in order to asses if under different maintenance requirements caused by changed ruminal Ph, bacteria would change their growth and 4) expand on the effect of LPS on the growth of other ruminal lactate producing bacteria.

In summary, all LPS treatments slowed down the growth and/or decreased the production of total OAs, acetate, and lactate in lactate -producing bacteria (*Se. ruminantium* HD4, *S. bovis* JB1), even though these species are phylogenetically and physiologically distinct, and did not affect the lactate-utilizing bacterium (*M. elsdenii* T81). Compared to E. COLI, RUM LPS exhibited greater rate of decrease of lactate production in *S. bovis* JB1, which indicates a potential biological difference on fatty acid content between ruminal and *E. coli* LPS. Our results, suggest that ruminal LPS would delay the development of ruminal acidosis by slowing down the growth and the accumulation of lactate from ruminal bacteria that use starch and produce lactate. Future directions should be focused on the determination of fatty acid composition of mixed ruminal lipid A and evaluation of the effects of C15–17 fatty acids on the growth of *Se. ruminantium* HD4 and *S. bovis* JB1.

## Materials and methods

All experimental procedures involving the animals used as donors of rumen fluid in the study were conducted under protocols approved by the University of Florida Institutional Animal Care and Use Committee (IACUC). Moreover, all methods were performed in accordance with the IACUC guidelines and regulations. The following study is reported in accordance with ARRIVE guidelines.

### Statistical analysis

Each treatment was tested with at least three biological replicates (n ≥ 3), for total sample size n = 12 per strain tested. Significance was declared at *P* ≤ 0.05, while tendency at 0.05 < *P* ≤ 0.10.

Logistic function was used to predict the growth rate (μ) and lag time (lag). According to^41^, the logistic function used was:

$$Y={y}_{0}+\frac{C}{\left(1+{exp}^{\left[4*\mu max*\frac{lag-t}{C}+2\right]}\right)}$$

Y is real ODt, y_0_ is initial OD_0_, μmax is maximum specific growth rate, and C is increase of OD from OD_0_ to OD_t_.

The predicted maximum specific growth rate and lag time were used as input for SAS analysis. The effects of treatment on maximum specific growth rate, lag time, and concentrations of ammonia, organic acids were analyzed using least-square analysis of variance (ANOVA) using the MIXED procedure of SAS.

The statistical model used was:

$${y}_{i}=\mu +{T}_{i}+{E}_{j}+{\varepsilon }_{ij}$$

where y is a dependent variable, μ is overall mean, ${T}_{i}$ is fixed effect of treatments,${E}_{j}$ is experimental run, and ${\varepsilon }_{ij}$ is the random error. Experimental run was considered as random effect.

### Fractionation of ruminal fluid

Ruminal fluid was obtained from a rumen-cannulated Holstein cow fed ad libitum a total mixed ration (DM basis: 60% whole plant corn silage, 12.5% ground corn, 13% citrus pulp, 12% soybean meal, and 2.5% mineral and vitamin mix). Approximately 3 h after morning feeding, ruminal contents were manually collected (7 L) and strained through four layers of cheesecloth into pre-warmed thermos bottles and promptly transported to the lab. The contents were strained again through two-layer cheesecloth, transferred into beakers, and immersed in ice for 15 min. The strained ruminal fluid (approximately 14 L) was centrifuged (Sorvall RC-5B Refrigerated Superspeed Centrifuge, DuPont Instruments® Wilmington, DE) three times in succession. First at 1000×*g* for 10 min, then the supernatant was collected and centrifuged again at 11,250×*g* for 20 min, then the bacteria pellet obtained, resuspended in Milli-Q water and centrifuged for a third time at 16,250×*g* for 20 min to obtain the pellet of bacteria that was later resuspended in Milli-Q water. Finally, bacterial pellets were transferred to pyrogen-free tubes, homogenized and diluted to 15 mL using Milli-Q water and stored in – 80 °C for later ruminal LPS extraction.

### Ruminal lipopolysaccharide extraction

A modified hot-phenol extraction was utilized to extract LPS from ruminal bacteria obtained from the rumen-cannulated cow as described previously^42,43^ but with minor modifications and validations described below. Briefly to isolate total LPS from ruminal fluid, the bacterial pellet was boiled at 100–110 °C using a heat block for 30 min followed by the addition of 50 mL Milli-Q water. The bacteria suspension was then treated with 50 mL of 90% phenol that was prewarmed at 68 °C for 30 min. The preparation was then placed in − 20 °C for 30 min to cool and centrifuged at 5000×*g* for 10 min. The aqueous (top) layer was then collected because it exhibits the greatest concentration of LPS after being tested with silver stain (Thermo Scientific™ Pierce™ Silver Stain Kit). The aqueous layer was then transported into a regenerated cellulose dialysis membrane (Fisherbrand™) for further dialysis against Milli-Q at 4 °C until phenol was not detectable at 260 nm in Milli-Q. Dialyzed samples were then treated with 5 mM MgCl_2_ followed by 20 μg/mL Dnase I (M0303s, New England Biolabs) for 2 h at 37 °C to degrade contaminating DNA. After, 20 μg/mL Rnase H (T3018, New England Biolabs) was added for 2 h at 37 °C, to degrade contaminating RNA and, last 30 mg/mL Proteinase K (Fisher BioReagents™ Proteinase K, Catalog No. BP1700-100) was added to remove protein contamination. The preparation was then lyophilized and crude LPS mass was determined. After lyophilization, dry samples were resuspended into 15 mL of Milli-Q water and centrifuged at 1110×*g* for 10 min to remove any solids. The supernatant was treated with 0.15 mL 50 mM acetic acid, 95% ethanol and transferred with glass Pasteur pipette into ultracentrifuge tubes (Quick-Seal® Round-Top Polypropylene Tube) and then spun for 8 h at 4 °C and 105,000×*g* in an ultracentrifuge (optima XE, Beckman Coulter Life Sciences, Indianapolis, Indiana). The supernatant was removed and LPS gels were resuspended in 2 mL of endotoxin-free water and lyophilized to determine the dry weight of pure LPS. To confirm the purity and normalization of ruminal-derived LPS, the final products were visualized with the Pierce™ Silver Stain Kit (Thermo Scientific™) in accordance with the manufacturer’s instructions. In all cases, the Pierce™ Silver Stain Kit indicated a purity identical to that of LPS purified from pure bacterial isolates.

### LPS stock preparation

The concentrations of *E. coli*-LPS (*Escherichia coli* O111:B4, L2630; Sigma-Aldrich Co., St. Louis, MO), ruminal-LPS and MIX-LPS were 200,000 EU (1 ng/mL = 10 EU based on the Sigma-Aldrich protocol).

All LPS stocks were prepared under anaerobic condition, in which 25 mg of *E. coli*-LPS were added to 62.5 mL sterile, anaerobic, nonpyrogenic water while flushing with CO_2_, to generate 0.4 mg/ mL of *E. coli*-LPS. The ruminal-LPS stock (25 mg) was resuspended in 2 mL sterile, nonpyrogenic water and sonicated for 20 min. After sonication, the ruminal LPS stock was prepared under anaerobic conditions and the final volume was brought to 62.5 mL in sterile, anaerobic, nonpyrogenic water to generate 0.4 mg/mL stock. For MIX-LPS stock, 13.5 mL (equal to 5.4 mg of *E. coli*-LPS) from *E. coli*-LPS stock was mixed with 13.5 mL (equal to 5.4 mg of ruminal-LPS) ruminal-LPS stock to generate 0.4 mg/mL of MIX-LPS. All LPS stocks were then filtered through a 0.45-μm followed by a 0.22-μm polyethersulfone (PES) membrane syringe filter (Celltreat, Pepperell, MA) into serum bottles that were previously flushed with CO2 and autoclaved. A 0.5 mL volume of 0.4 mg/mL *E. coli*-, ruminal- and MIX- LPS stock contained 200,000 EU of LPS. We chose the 0.4 mg/mL concentration assuming that ruminal LPS would equate *E. coli* LPS by weight. This assumption was made to make all of the doses similar in weight, volume, and endotoxicity. To validate the endotoxicity of the MIX stock solution, we performed limulus amebocyte lysate assay, which exhibited endotoxicity close to 200,000 EU.

### Media

The basal medium contained 240 mg of K_2_HPO_4_, 240 mg of KH_2_PO_4_, 480 mg of (NH_4_)_2_SO_4_, 480 mg of NaCl, 100 mg of MgSO_4_·7H_2_O, 64 mg of CaCl_2_·2H_2_O, 600 mg cysteine hydrochloride, 1 g of trypticase peptone (product 212750; BD), and 0.5 g of yeast extract (product 212750; BD) per liter^44^; Ph 6.5; autoclaved (121 °C, 15 min) to remove O_2_ and cooled under O_2_-free CO_2_. Sodium carbonate (4 g/L) was added as a buffer. Resazurin was added as a redox indicator. Growth substrates were anaerobically prepared and introduced to the basal medium under sterile conditions. Glucose (20 mM final concentration) was added as growth substrate for *S. bovis* JB1 and *Se. ruminantium* HD4 and 50 mM lactate (final concentration) was added as a growth substrate for *M. elsdenii* T81. All media and media additives (glucose or lactate) were based on previous peer reviewed studies of pure cultured ruminal bacteria^44^ and were prepared at the same time to reduce variability between runs.

### Organisms

Lactate producing bacteria *Selenomonas ruminantium* HD4 (chain of custody: Herbert J. Strobel, Michael D. Flythe) and *Streptococcus bovis* JB1 (chain of custody: James B. Russell, Michael D. Flythe), and lactate utilizing bacteria *Megasphaera elsdenii* T81 (chain of custody: Paul J. Weimer, Michael D. Flythe) were obtained from the stock culture collection maintained at the Forage-Animal Production Research Unit, ARS, USDA on the University of Kentucky campus. All isolates were verified for purity via Gram-stain and microscopy^45^. Preliminary growth curve analyses were conducted to determine the lag, log, and stationary phases of each strain (data not shown).

### Treatments and measurement of bacterial growth

Strains were inoculated into 10 mL growth media (lactate-producers: basal medium plus 20 mM glucose; lactate-utilizers: basal medium plus 50 mM lactate) and incubated at 39 °C overnight. The optical density (OD, absorbance 600 nm) of each overnight culture was recorded to determine the inoculum for the growth curve experiments. One hundred Μl of *S. bovis* JB1, 100 µL *Se. ruminantium* HD4, or 500 µL *M. elsdenii* T81 were added to basal medium with 0.5 mL LPS treatment or control. Treatments were (i) CTRL, control group (LPS-free anaerobic water); (ii) RUM, ruminal-LPS (0.4 mg/ mL ruminal LPS); (iii) E. COLI, *E. coli*-LPS (0.4 mg/mL *E. coli*-LPS); and (iv) MIX, 1:1 *E. coli*: Ruminal-LPS (0.4 mg/mL MIX-LPS). The concentration of all three LPS (RUM, E. COLI, MIX) was chosen based on previous studies in which LPS concentration was measured in cows with SARA (200,000 EU)^46^. All experimental tubes were inverted to mix, flame sterilized, and 2 mL was removed for baseline fermentation end products (NH_3_-N, and organic acids) and the initial optical density (OD_0_). All strains were grown anaerobically under O_2_-free CO_2_ in Hungate tubes and incubated at 39 °C without shaking. Optical densities (ODt) were recorded hourly except in the case of *S. bovis* JB1, for which measurements were collected every 30 min, until bacterial growth reached a plateau. Once bacterial growth reached mid-exponential phase, 2 mL culture medium was collected into Eppendorf tubes, clarified by centrifugation (15,000×*g*, 2 min), and frozen at  − 20 °C for later determination of the fermentation end products (NH_3_-N, and organic acids). Samples for fermentation end products were collected at mid-exponential phase to represent continuous fermentation conditions as it happens in the rumen (Figs. 1, 2, 3).

### Fermentation end product analyses

Samples of the media were thawed, clarified by centrifugation (15,000×*g*, 2 min), and the ammonia concentrations were determined by the phenolic acid/hypochlorite method^47^. Volatile fatty acids, lactate, and soluble sugar concentrations were quantified by HPLC (Dionex, Sunnyvale, CA, USA). The column (Aminex HP-87H, Bio-Rad, Hercules, CA) was operated at 50 °C, with a 0.4 mL/min flow rate and aqueous H_2_SO_4_ (0.17 N) mobile phase. A refractive index detector (Shodex/Showa Denko, Kanagawa, Japan) and a UV detector (Dionex, Sunnyvale, CA, USA) were used in tandem to detect eluting compounds.

## Data Availability

The datasets used and/or analyzed during the current study are available from the corresponding author on reasonable request.
